# Archaeal Phospholipid Biosynthetic Pathway Reconstructed in *Escherichia coli*

**DOI:** 10.1155/2012/438931

**Published:** 2012-05-09

**Authors:** Takeru Yokoi, Keisuke Isobe, Tohru Yoshimura, Hisashi Hemmi

**Affiliations:** Department of Applied Molecular Bioscience, Graduate School of Bioagricultural Sciences, Nagoya University, Furo-cho, Chikusa-ku, Nagoya, Aichi 460-8601, Japan

## Abstract

A part of the biosynthetic pathway of archaeal membrane lipids, comprised of 4 archaeal enzymes, was reconstructed in the cells of *Escherichia coli*. The genes of the enzymes were cloned from a mesophilic methanogen, *Methanosarcina acetivorans*, and the activity of each enzyme was confirmed using recombinant proteins. *In vitro* radioassay showed that the 4 enzymes are sufficient to synthesize an intermediate of archaeal membrane lipid biosynthesis, that is, 2,3-di-*O*-geranylgeranyl-*sn*-glycerol-1-phosphate, from precursors that can be produced endogenously in *E. coli*. Introduction of the 4 genes into *E. coli* resulted in the production of archaeal-type lipids. Detailed liquid chromatography/electron spray ionization-mass spectrometry analyses showed that they are metabolites from the expected intermediate, that is, 2,3-di-*O*-geranylgeranyl-*sn*-glycerol and 2,3-di-*O*-geranylgeranyl-*sn*-glycerol-1-phosphoglycerol. The metabolic processes, that is, dephosphorylation and glycerol modification, are likely catalyzed by endogenous enzymes of *E. coli.*

## 1. Introduction

 Archaeal membrane lipids are very specific to the organisms in the domain Archaea and have structures that are distinct from those of bacterial/eukaryotic lipids [1–3]. Although they are, essentially, analogues of glycerolipids from bacteria or eukaryotes, they have specific structural features as follows: (1) hydrocarbon chains of archaeal lipids are multiply-branched isoprenoids typically derived from (all-*E*) geranylgeranyl diphosphate (GGPP), while linear acyl groups are general in bacterial/eukaryotic lipids; (2) the isoprenoid chains are linked with the glycerol moiety with ether bonds, while ester bonds are general in bacterial/eukaryotic lipids; (3) the glycerol moiety of archaeal lipids is derived from *sn*-glycerol-1-phosphate (G-1-P), which is the enantiomer of *sn*-glycerol-3-phosphate, the precursor for bacterial/eukaryotic glycerolipids; (4) dimerization of membrane lipids by the formation of carbon-carbon bonds between the *ω*-terminals of hydrocarbon chains, which generates macrocyclic structures such as caldarchaeol-type lipids with a typically 72-membered ring, is often observed in thermophilic and methanogenic archaea. These characteristics affect the properties of membranes formed with the lipids. In general, the permeability of membranes composed of archaeal lipids is lower than that of membranes that consist of bacterial/eukaryotic lipids [4, 5]. Moreover, the structural differences between archaeal and bacterial/eukaryotic lipids are believed to cause their black-and-white distribution between these domains without exception (the “lipid divide”) [6, 7]. This hypothesis is based on the idea that a membrane composed of both archaeal- and bacterial/eukaryotic-type lipids is disadvantageous to the organism, compared with membranes composed of one type. Although this hypothesis is attractive, no proof of it has been reported so far. To obtain proof of this hypothesis, two lines of experiments can be designed. One is to compare the physical properties of artificial membranes prepared with the archaeal- and/or bacterial/eukaryotic-type lipids. A few studies of this type have been done [8, 9]. Shimada and Yamagishi [9] recently reported that hybrid liposomes constructed from both archaeal- and bacterial-type lipids were generally more stable (impermeable) than they had expected. Based on these results, they concluded that the common ancestor of life (and the origin of eukaryotes supposedly formed by the fusion of archaea- and bacteria-like cells) might have had such hybrid lipid membranes, but they did not explain how the lipid divide occurred. The other, more straightforward line of experiments is to generate an organism that synthesizes both archaeal- and bacterial/eukaryotic-type membrane lipids and therefore has hybrid membranes. If the hybrid membranes are disadvantageous for the organism because of properties such as stability, permeability, and fluidity, the organism may lower viability or become susceptible to stresses such as heat and osmotic shock. Lai et al. recently reported the construction of such an organism, although they did not determine its phenotypes [10]. In their study, phospholipid biosynthetic genes from a hyperthermophilic archaeon *Archaeoglobus fulgidus* were introduced into *Escherichia coli*. The authors demonstrated the synthesis of precursors for archaeal membrane lipids, that is, 3-*O*-geranylgeranyl-*sn*-glycerol-1-phosphate (GGGP) and 2,3-di-*O*-geranylgeranyl-*sn*-glycerol-1-phosphate (DGGGP), in the recombinant *E. coli,* based on the detection of corresponding alcohols from the lipid extract from the cells after phosphatase treatment. However, it was still unclear whether the archaeal-type lipids produced in the cells actually acted as the structural components of a membrane bilayer, because the authors did not show the intact structures of the lipids. Moreover, they did not describe the level of production of the archaeal-type lipids, which is also important to the evaluation of their effects on the membranes of *E. coli*.

**Figure 1:**
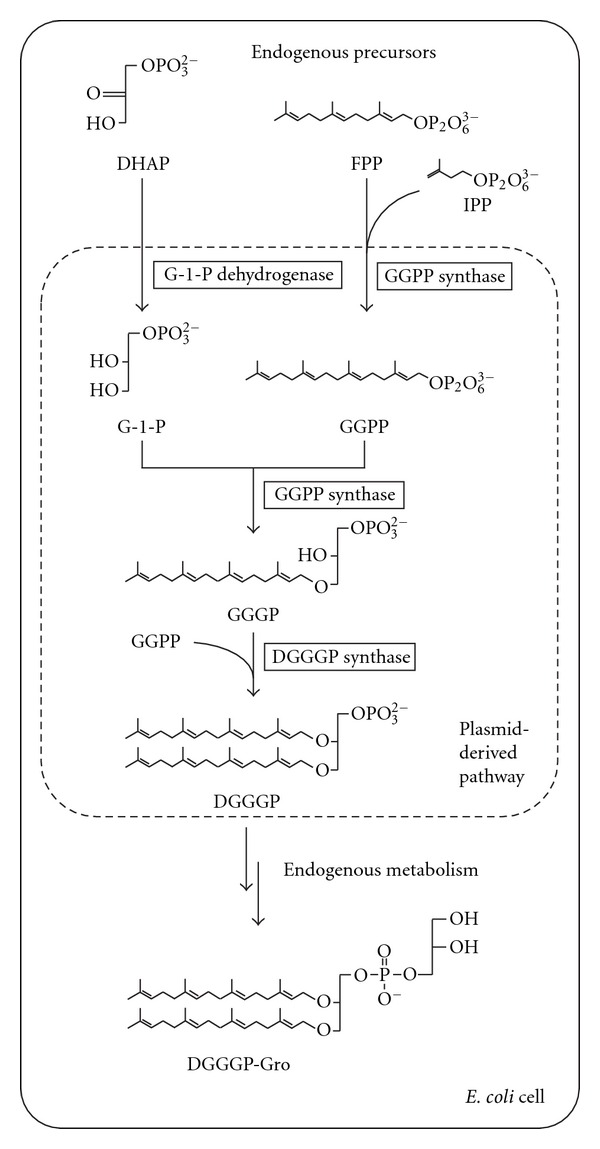
Biosynthetic pathway of archaeal-type lipids reconstructed in *E. coli*.

 In the present study, we reconstructed a part of the biosynthetic pathway of an archaeal phospholipid (Figure 1), which consisted of G-1-P dehydrogenase, GGPP synthase, GGGP synthase, and DGGGP synthase from a mesophilic methanogenic archaeon, *Methanosarcina acetivorans*, in *E. coli*. These enzymes can synthesize DGGGP from the endogenous precursors of isoprenoid in *E. coli*, that is, (all-*E*) farnesyl diphosphate (FPP), isopentenyl diphosphate (IPP), and dihydroxyacetone phosphate (DHAP). In addition, the enzymes from the mesophile were expected to have optimal activities at the growth temperature of *E. coli*, which would lead to high-level production of the archaeal phospholipid precursor and its derivatives. We evaluated the total amount and intact structures of the archaeal-type lipids extracted from the cells by liquid chromatography/electron spray ionization-mass spectrometry (LC/ESI-MS) analysis and showed that DGGGP was metabolized by enzymes endogenous to *E. coli*.

## 2. Materials and Methods

### 2.1. Materials

LKC-18F precoated, reversed-phase, thin-layer chromatography plates were purchased from Whatman, UK. FPP was donated by Drs. Kyozo Ogura and Tanetoshi Koyama, Tohoku University. [1-^14^C]IPP was purchased from American Radiolabeled Chemicals, USA. All other chemicals were of analytical grade.

### 2.2. General Procedures

Restriction enzyme digestions, transformations, and other standard molecular biological techniques were carried out as described by Sambrook et al. [11].

### 2.3. Cultivation of *M. acetivorans*

The *M. acetivorans* C2A (JCM 12185) strain was provided by the Japan Collection of Microorganisms (JCM), RIKEN BRC, through the Natural Bio-Resource Project of the MEXT, Japan. The mesophilic methanogenic archaeon was cultivated in a JCM 385 *Methanosarcina acetivorans* medium at 37°C and harvested at the log phase.

### 2.4. Construction of Plasmids Containing Phospholipid Biosynthetic Genes from *M. acetivorans*

The genome of *M. acetivorans* was extracted from the cells using a DNA extraction kit, ISOPLANT II (Nippon Gene). Each of the hypothetical genes for archaeal phospholipid biosynthesis, that is, *MA3686*, *MA0606*, *MA3969*, and *MA0961*, was amplified using the primers shown in Table 1, using the genome of *M. acetivorans* as a template, and using KOD DNA polymerase (Toyobo, Japan). The amplified DNA fragment was digested by restriction enzymes that recognize the sites in the primers and then inserted into the pBAD18 vector cut with the same restriction enzymes to construct the plasmid for expression of each archaeal enzyme, that is, pBAD-MA3686, pBAD-MA0606, pBAD-MA3969, and pBAD-MA0961.

For the construction of plasmids for expression of multiple archaeal genes, an In-Fusion Advantage PCR cloning kit (Takara, Japan) was used according to the manufacturer's instructions. The *MA0961* gene was amplified using the primers shown in Table 1 and pBAD-MA0961 as a template. By the action of the In-Fusion enzyme, the amplified fragment was inserted into the plasmid pBAD-MA0606, which had been digested with *Sal*I, to construct the plasmid pBAD-ALB2. Next, the *MA3969* gene, which was amplified using the primers in Table 1 and pBAD-MA3969 as a template, was inserted into pBAD-ALB2 digested with *Sal*I to construct pBAD-ALB3. The plasmid was then digested with *Sal*I, and the *MA3686* gene, amplified using the primers in Table 1 and pBAD-MA3686 as a template, was inserted to construct pBAD-ALB4.

### 2.5. Recombinant Expression of the Archaeal Enzymes

*E. coli* Top10, transformed with each plasmid containing a homologous gene for archaeal phospholipid biosynthesis, that is, pBAD-MA0606, pBAD-MA3969, pBAD-MA0961, pBAD-MA3686, or pBAD-ALB4, was cultivated at 37°C in 250 mL LB medium supplemented with 100 mg/L ampicillin. When the optical density at 660 nm of the culture reached 0.5, then 0.02% of L-arabinose was added for induction. After an additional 16 h incubation, the cells were harvested and disrupted by sonication in 5 mL of 100 mM 3-(*N*-morpholino)propanesulfonic acid (MOPS)-NaOH buffer, pH 7.0. The homogenates were centrifuged at 24,000 g for 30 min to recover the supernatants as a crude extract, which was used for enzyme assay.

### 2.6. *In Vitro* Assay for the Biosynthesis of Phospholipid Precursors

The assay mixture for prenyltransferases contained, in a final volume of 200 *μ*L 0.2 nmol of [1-^14^C]IPP (2.04 GBq/mmol), 1 nmol of FPP, 2.0 *μ*mol of MgCl_2_, 20 *μ*mol of MOPS-NaOH, pH 7.0, and suitable volumes of the crude extracts from *E. coli* containing pBAD-MA0606, pBAD-MA3969, or pBAD-MA0961. *α*-Glycerophosphate (racemic mixture) was added only to the mixtures containing MA3969.

The assay mixture for G-1-P dehydrogenase contained, in a final volume of 200 *μ*L, 0.2 nmol of [1-^14^C]IPP (2.04 GBq/mmol), 1 nmol of FPP, 2.0 *μ*mol of MgCl_2_, 20 *μ*mol of MOPS-NaOH, pH 7.0, and suitable volumes of the crude extracts from *E. coli *containing pBAD-MA0606, pBAD-MA3969, or pBAD-MA3686. If needed, 200 nmol of *α*-glycerophosphate or DHAP was added to the mixture.

In a final volume of 200 *μ*L, the assay mixture for the 4 archaeal enzymes simultaneously expressed in *E. coli* contained, 0.2 nmol of [1-^14^C]IPP (2.04 GBq/mmol), 1 nmol of FPP, 200 nmol of DHAP, 2.0 *μ*mol of MgCl_2_, 20 *μ*mol of MOPS-NaOH, pH 7.0, and a suitable volume of the crude extract from *E. coli* containing pBAD-ALB4.

After incubation at 37°C for 30 min, the reaction was stopped by chilling in an ice bath. 200 *μ*L of water saturated with NaCl was added to the mixture, and then the products were extracted with 600 *μ*L of 1-butanol saturated with NaCl-saturated water. They were treated with acid phosphatase according to the method of Fujii et al. [12], and the hydrolysates were extracted with *n*-pentane and analyzed by reversed-phase, thin-layer chromatography (TLC) using a precoated plate LKC-18F developed with acetone/H_2_O (9 : 1). The distribution of radioactivity was detected using a BAS2000 bioimaging analyzer (Fujifilm, Japan). The authentic samples were prepared as described in our previous reports, using the enzymes from *Sufolobus acidocaldarius* and *S. solfataricus* [13].

### 2.7. Lipid Isolation from *E. coli* Harboring pBAD-ALB4

Lipid was extracted from 2 g of wet cells of *E. coli *harboring pBAD-ALB4, cultured as described above, except that induction with l-arabinose was performed for 18 h. The cells were dissolved with 15 mL of 1-butanol/75 mM ammonium water/ethanol (4 : 5 : 11). The mixture was heated to 70°C and shaken vigorously for 1 min. It was heated again at 70°C for 20 min and shaken vigorously again for 1 min. After cooling to room temperature, the mixture was centrifuged at 1,000 *g* for 10 min. The supernatant was recovered and dried under a stream of nitrogen at 55°C. The dried residue was then dissolved with 7.2 mL of 1-butanol/methanol/0.5 M acetate buffer, pH 4.6 (3 : 10 : 5). Lipids in the mixture were extracted with 3 mL *n*-pentane and dried under a stream of nitrogen at 55°C. The dried residue was then redissolved in 1 mL of methanol/2-propanol (1 : 1).

### 2.8. LC/ESI-MS Analysis

ESI-MS was performed with an Esquire 3000 ion trap system (Bruker Daltonics, USA). MS-parameters used were as follows: sheath gas, N_2_ of 30 psi; dry gas, N_2_ of 7.0 L*·*min^−1^ at 320°C; scanning range, 50–1,000 *m/z*; scan speed, 13,000 *m/z·*sec^−1^; ion charge control target, 50,000 or 20,000; maximum accumulation time, 100 ms; averages, 10; rolling averaging, 2. The system was equipped with an Agilent 1100 Series HPLC system (Agilent Technologies, USA) using UV detection at 210 nm and COSMOSIL Packed Column 5C_18_-AR-II (2.0 × 150 mm, Nacalai, Japan). The mobile phase consisted of methanol/100 mg*·*L^−1^ sodium acetate (9 : 1) or methanol/120 mg*·*L^−1^ potassium acetate (9 : 1). The flow rate was 0.2 mL·min^−1^.

### 2.9. Sodium Periodate Treatment

For sodium periodate treatment of 2,3-di-*O*-geranylgeranyl-*sn*-glycero-1-phosphoglycerol (DGGGP-Gro), the peak fraction from HPLC contained about 1 nmol of the mixture of DGGGP-Gro and 2,3-di-*O*-geranylgeranyl-*sn*-glycerol (DGGGOH), and 1 *μ*mol sodium periodate was added to 1 mL of 1-butanol/75 mM ammonium water/ethanol (4 : 5 : 11). The mixture was reacted at 25°C for 1 h in the dark. The reaction was stopped by adding 1.5 *μ*mol of glycerol. After 15 min, the product was extracted by 1 mL of *n*-pentane and dried with N_2_. The dried residue was dissolved with 100 *μ*L of methanol/2-propanol (1 : 1) and analyzed by LC/ESI-MS.

## 3. Results and Discussion

**Figure 2:**
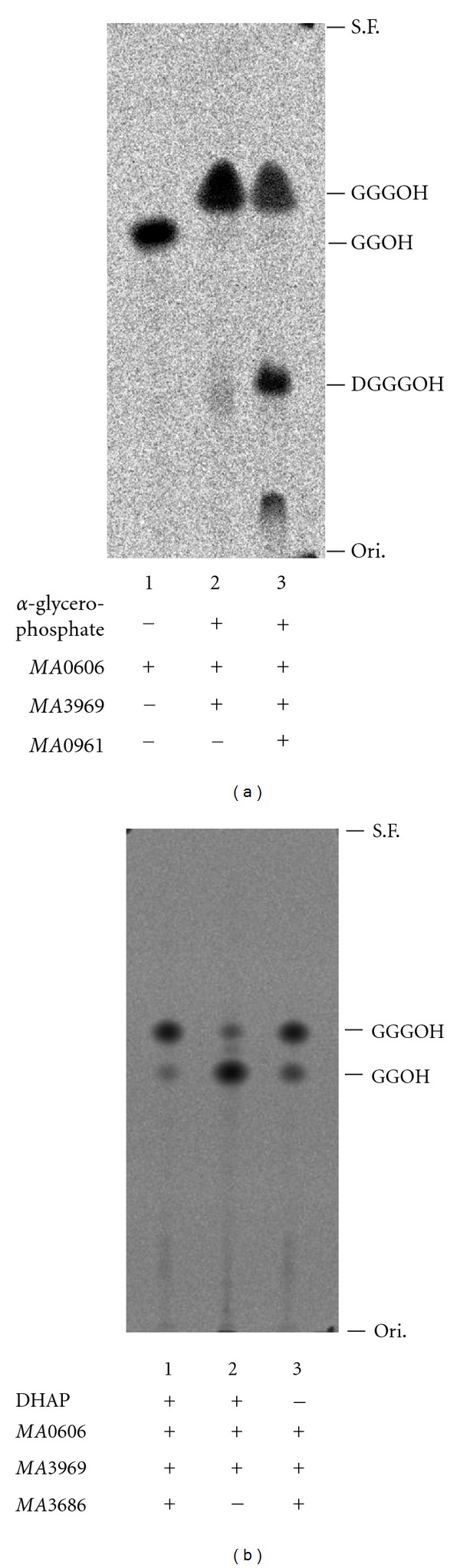
*In vitro* assay of the archaeal enzymes for phospholipid biosynthesis. (a) Thin-layer radiochromatogram of the dephosphorylated products from the reactions with recombinant *M. acetivorans* GGPP synthase, GGGP synthase, and/or DGGGP synthase. The enzyme assays were performed using FPP, [^14^C]IPP, and *α*-glycerophosphate as the substrates. (b) Thin-layer radiochromatogram of the products from the reaction with recombinant *M. acetivorans* G-1-P dehydrogenase, coupled with GGPP synthase and GGGP synthase. FPP, [^14^C]IPP, and DHAP were used as the substrates. S.F., solvent front; Ori., origin.

We first searched for and cloned the genes from a mesophilic, methanogenic archaeon, *M. acetivorans, *which encoded the closest homologues of the enzymes involved in the biosynthesis of archaeal membrane lipids. The homologue of G-1-P dehydrogenase, which shows 59% sequential identity with G-1-P dehydrogenase from *Methanothermobacter thermautotrophicus* [14], is encoded in the gene *MA3686*. The closest homologue of GGPP synthase, with 39% identity with the enzyme from *S. acidocaldarius* [15], is encoded in *MA0606*. The GGGP synthase homologue with 57% identity with the enzyme from *M. thermautotrophicus* [16] is encoded in *MA3969*. The closest homologue of DGGGP synthase, with 31% identity with the enzyme from *S. solfataricus* [13], is encoded in *MA0961*. Each of the genes, *MA3686*, *MA0606*, *MA3969*, and *MA0961*, was recombinantly expressed in *E. coli*. The cells of *E. coli* were disrupted and centrifuged to recover the supernatant as the crude extract. Then the enzyme activity in the crude extract was confirmed by radio-TLC assay. As shown in Figure 2(a), incubation of the crude extract from *E. coli *expressing *MA0606* with FPP and [^14^C]IPP yielded a radiolabeled hydrophobic product, and treatment of the product with acid phosphatase produced a compound that comigrated with authentic (all-*E*) geranylgeraniol on a reversed-phase TLC plate (*R_f_* = 0.60). Addition of the crude extract from *E. coli* expressing *MA3969* and *α*-glycerophosphate to the reaction mixture resulted in the movement of the radiolabeled spot on TLC. The new spot (*R_f_* = 0.68) comigrated with authentic 3-*O*-geranylgeranyl-*sn*-glycerol (GGGOH). The movement did not occur in the absence of *α*-glycerophosphate (data not shown). When the crude extract from *E. coli* expressing *MA0961* was additionally mixed, new radiolabeled spots (*R_f_* = 0.34 and 0.12) emerged on TLC, accompanied by diminishing radioactivity of the other spot. The spot with an *R_f_* of 0.34 comigrated with authentic DGGGOH. These results indicate that *MA0606*, *MA3969*, and *MA0961* encode, as expected from their homologies, GGPP synthase, GGGP synthase, and DGGGP synthase, respectively. The spot with an *R_f_* of 0.12 was considered to have originated from an unknown modification of DGGGP catalyzed by enzymes contained in the crude extracts. To confirm G-1-P dehydrogenase activity in the crude extract of *E. coli* expressing *MA3686*, the extract was incubated with the crude extracts containing *M. acetivorans *GGPP synthase and GGGP synthase, DHAP, FPP, and [^14^C]IPP. The hydrophobic product was extracted, treated with phosphatase, and analyzed by TLC, giving a main spot that comigrated with GGGOH (Figure 2(b)). After removal of the crude extract of *E. coli *expressing *MA3686* from the reaction mixture, the GGGOH spot became thinner, and a spot that comigrated with geranylgeraniol became the major spot. This result shows that *MA3686* encodes G-1-P dehydrogenase. In contrast, removal of DHAP from the mixture did not change the TLC profile of the products, suggesting that a sufficient amount of DHAP existed in the reaction mixture, which contained cell extracts from *E. coli*. It is noteworthy that a small amount of GGGOH appears to be synthesized even in the absence of *M. acetivorans *G-1-P dehydrogenase. It is possible that the enzyme has only low affinity for *sn*-glycerol-3-phosphate, as has been reported with G-1-P-specific archaeal homologues [17–19].

**Figure 3:**
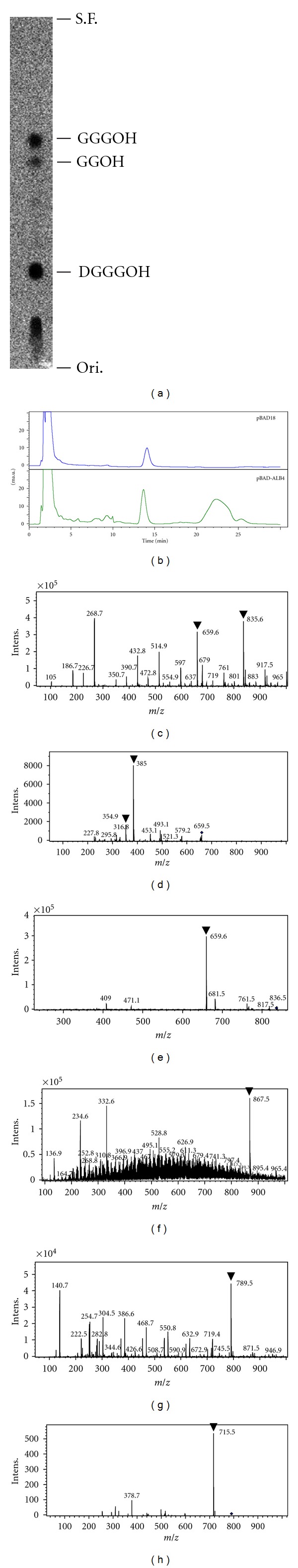
Radio-TLC and LC/ESI-MS analyses of the archaeal-type lipids synthesized in *E. coli*. (a) Crude extract from *E. coli* harboring pBAD-ALB4 was incubated with FPP, [^14^C]IPP, and DHAP. Reversed-phase radio-TLC analysis of the products was performed after dephosphorylation. S.F., solvent front; Ori., origin. (b) LC profiles of lipids extracted from *E. coli *harboring pBAD-ALB4 (lower) or its parent plasmid, pBAD18 (upper). (c) Positive ESI-MS ion spectrum of a peak in (b) around 22 min. (d) and (e) MS/MS analyses of the ions in (c), with *m/z* of 659.6 and 835.6, respectively. (f) Positive ESI-MS ion spectrum of the LC peak corresponding with that analyzed in (c). Exclusively for this analysis, the elution buffer was changed from sodium based to potassium based. (g) Negative ESI-MS ion spectrum of a peak in (b) around 22 min. (h) MS/MS analysis of the ion in (g), with an *m/z* of 789.5.

We next constructed a plasmid vector containing the 4 archaeal genes, which formed an artificial operon in the order *MA0606*-*MA0961*-*MA3969*-*MA3686*, to reconstruct the biosynthetic pathway of archaeal phospholipid in *E. coli*. The activities of the enzymes were confirmed by *in vitro* radio-TLC assay. The cell extract from recombinant *E. coli *expressing the 4 archaeal genes showed activities related to the formation of DGGGP from DHAP, IPP, and FPP *in vitro* (Figure 3(a)), which indicated that the enzymes from *M. acetivorans*, that is, G-1-P dehydrogenase, GGPP synthase, GGGP synthase, and DGGGP synthase, were all expressed in the cells. In addition, a radioactive spot with a lower *R_f_* value (~0.1) was observed. This spot probably corresponded with the one with an *R_f_* of 0.12 observed in Figure 2(a). Because these spots accompanied the formation of DGGGP and because reaction mixtures for these assays contained cell extracts from *E. coli*, they were considered to arise from an unknown derivative of DGGGP, which might be formed through endogenous metabolic pathways in *E. coli*.

Thus, we extracted lipids from the recombinant *E. coli* cells to confirm *in vivo* synthesis of the archaeal phospholipid precursors or their derivatives. The results of LC/ESI-MS analysis of the extract from *E. coli* containing pBAD-ALB4 showed a relatively broad LC peak of A_210_, which eluted from the column at ~22 min (Figure 3(b)). This peak was absent in the analysis of the extract from *E. coli* containing the parent plasmid pBAD18. Specific ion peaks with *m/z* of 659.6 and 835.6 were detected through MS analysis of the peak in the positive ion mode (Figure 3(c)). These ions had similar but slightly different peak retention times, so the smaller ion was unlikely derived from fragmentation of the larger one. The smaller ion with *m/z* of 659.6 corresponded with [DGGGOH+Na]^+^. As shown in Figure 3(d), MS/MS analysis of the ion gave a fragment ion with an *m/z* of 385.0, which corresponded with [GGGOH+Na-2H]^+^. In addition, a smaller fragment ion with an *m/z* of 354.9, which corresponded with [GGGOH+Na-CH_2_O]^+^, was detected. The fragmentation pattern supported the idea that the peak in Figure 3(b) contained DGGGOH, which probably synthesized by the action of the exogenous archaeal enzymes and endogenous phosphatases in *E. coli*. On the other hand, the MS/MS analysis of the larger ion with *m/z* of 835.6 found a fragment ion with an *m/z* of 659.6, suggesting that the parent ion contained the DGGGOH structure (Figure 3(e)). The MS/MS/MS analysis of the fragment ion with an *m/z* of 659.6 yielded fragment ions similar to those observed in Figure 3(d) (data not shown). We therefore presumed that the ion peak with an *m/z* of 835.6 was derived from the cationic bisodium salt of the phosphatidylglycerol-type derivative of DGGGP (DGGGP-Gro). To confirm this idea, the elution buffer for LC/ESI-MS was changed from one containing sodium acetate to one containing potassium acetate, and the same lipid extract was analyzed. As a result, an ion with *m/z* of 867.5, which corresponded well with that expected for the cationic bis-potassium salt of DGGGP-Gro, was detected instead (Figure 3(f)). In addition, MS analysis of the ion shown in Figure 3(b), in the negative ion mode, yielded an ion with *m/z* of 789.5, which corresponded with [DGGGP-Gro]^−^ (Figure 3(g)). MS/MS analysis of the ion showed a fragmentation ion with an *m/z* of 715.5, which is consistent with [DGGGP]^−^ (Figure 3(h)).

**Figure 4:**
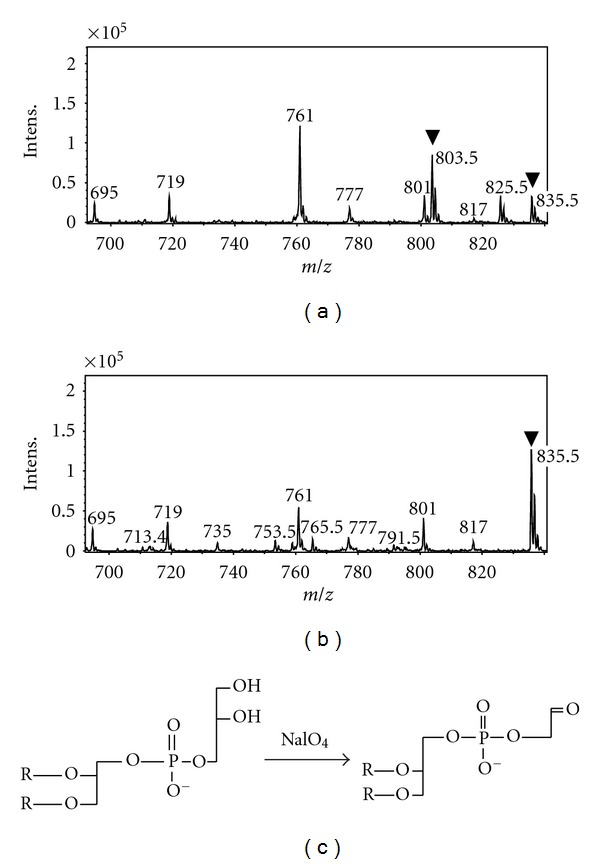
Sodium periodate treatment of DGGGP-Gro. (a) Positive ion spectrum from LC/ESI-MS analysis of the archaeal-type phospholipid postperiodate treatment and (b) preperiodate treatment. (c) The scheme of periodate treatment of DGGGP-Gro. R represents a geranylgeranyl group.

Moreover, we recovered the LC peak in Figure 3(b), which probably contained DGGGP-Gro, and treated the phospholipid with sodium periodate to confirm the structure of the polar head group. LC/ESI-MS analysis of the treated lipid with the elution buffer containing sodium acetate gave a positive ion with an *m/z* of 803.5 (Figure 4(a)), which was absent in the analysis of the untreated sample (Figure 4(b)). The emergence of this ion seemed to accompany the decline of the ion with an *m/z* of 835.5. The *m/z* of 803.5 corresponded well with the cationic bisodium salt of DGGGP modified with glycoaldehyde (2,3-di-*O*-geranylgeranyl-*sn*-glycero-1-phosphoglycoaldehyde), which had been expected as the product of the sodium periodate treatment of DGGGP-Gro (Figure 4(c)).

These results show that DGGGP, which should be synthesized from the precursors in *E. coli* cells by the action of the 4 exogenous archaeal enzymes, has been metabolized by endogenous *E. coli* enzymes to yield DGGGP-Gro. It is unclear whether the radioactive TLC spots with of ~0.1, observed in Figures 2(a) and 3(a), are derived from DGGGP-Gro. The archaeal-type phospholipid probably acts as a component of membranes in *E. coli*. Modification of phospholipids with glycerol is usual in *E. coli*, which produces phosphatidylglycerol as a major component of membrane phospholipids [20]. However, the most common phospholipid in the bacterium is phosphatidylethanolamine. The biosynthesis of these phospholipids starts from the cytidylation of phosphatidic acid, which yields CDP-diacylglycerol [21]. *sn*-Glycerol-3-phosphate or L-serine is then transferred to form phosphatidyl-*sn*-glycero-3-phosphate or phosphatidyl-L-serine, respectively. Dephosphorylation of the former intermediate yields phosphatidylglycerol, while decarboxylation of the latter yields phosphatidylethanolamine. If the formation of DGGGP-Gro proceeds through this pathway, the cytidyltransferase, *sn*-glycerol-3-phosphate transferase, and phosphatase of *E. coli* must accept the archaeal-type phospholipid as the substrate. However, the addition of CTP to the reaction mixture of the *in vitro* radio-TLC assay did not intensify the spot with an *R_f_* of ~0.1 (data not shown). In contrast, the fact that DGGGP modified with ethanolamine (or serine) was not detected in the LC/ESI-MS analyses suggested that the L-serine transferase did not accept the archaeal-type substrate. In fact, *E. coli *phosphatidylserine synthase, which belongs to an enzyme superfamily different from that which includes archaeal phosphatidylserine synthases, reportedly does not accept CDP-activated DGGGOH [22]. If the cytidylation-dependent pathway does not work, which seems more likely, the inner membrane-periplasmic phosphoglyceroltransferase system [23, 24] may transfer the *sn*-1-phosphoglycerol group from the 6-(glycerophospho)-D-glucose moiety of osmoregulated periplasmic glucans “membrane derived oligosaccharides”, or their lipid-linked precursors, to DGGGOH to yield DGGGP-Gro directly.

It should be noted that the growth rate of *E. coli* harboring pBAD-ALB4 was almost identical to that of *E. coli* harboring pBAD18 (data not shown). This fact suggests that the production of archaeal-type glycerolipids, which differ from endogenous bacterial ones in hydrocarbon structures and in chirality of the glycerol moiety, does not strongly affect the viability of *E. coli*. The total amount of archaeal-type lipids extracted from *E. coli* cells, which was estimated by comparing the area of the LC peak at A_210_ with that of known amounts of GGPP, was only ~60 *μ*g/g of wet cells. In addition, the archaeal-type lipids detected in this work, that is, DGGGP-Gro and DGGGOH, still retained double bonds in their hydrocarbon chains, which are rarely found in mature archaeal lipids. Therefore, it appears to be too early to conclude that the coexistence of archaeal and bacterial lipids is not disadvantageous for the organisms.

## Figures and Tables

**Table 1:** Primers used for plasmid construction.

Primers	Sequences (restriction enzymes that recognize the underlined sites)
For the construction of pBAD-MA0606	

ma0606fw	GTAAAGAATTCAGATATAAGGAAATAGATGTGATGCTTATGATGCTTAT (*Eco*RI)
ma0606rv	GGTATTTCTAGATTGTATCCTTATTTTTCAGTATTCCCTTGCAATCA (*Xba*I)

For the construction of pBAD-MA3969	

ma3969fw	TTATATAGCTAGCTATTAAAAATAAGGATAATTAATGCAGGTGGAAGCACACCT (*Nhe*I)
ma3969rv	GAAATGTCGACGATATATCTCCTTTTATTTTTAGCTTTTTATAGCTGATA (*Sal*I)

For the construction of pBAD-MA0961	

ma0961fw	ATAGAATTCAAGAAGATTATAATGTCTGCCGGAATAC (*Eco*RI)
ma0961rv	GATTCTAGATCATACACCGGCAATGAAAG (*Xba*I)

For the construction of pBAD-MA3686	

ma3686fw	CTATTGAGCTCAAATAAAAGGAGATATATCATGAAATTGACCATCAATA (*Sac*I)
ma3686rv	ATATTGGTACCATCTATTTCCTTATATCTTCAACTTATGACCTTTGTGA (*Kpn*I)

For the construction of pBAD-ALB2 by amplification of *ma0961 *	

alb2fw	ACAATCTAGAGTCGAAGGAAGATTATAATGTCTGCCGGAATAC
alb2rv	ATGCCTGCAGGTCGACTCATACACCGGCAATGAAAG (*Sal*I)

For the construction of pBAD-ALB3 by amplification of *ma3969 *	

alb3fw	CGGTGTATGAGTCGAAAGGAGTAATTAATGCAGGTGGAAGCACACCT
alb3rv	ATGCCTGCAGGTCGACTTAGCTTTTTATAGCTGATA (*Sal*I)

For the construction of pBAD-ALB4 by amplification of *ma3686 *	

alb4fw	AAAAAGCTAAGTCGAAAGGAGATATATCATGAAATTGACCATCAATA
alb4rv	ATGCCTGCAGGTCGACTCAACTTATGACCTTTGTGA (*Sal*I)
